# Extracorporeal support for pulmonary resection: current indications and results

**DOI:** 10.1186/s12957-016-0781-0

**Published:** 2016-02-02

**Authors:** Petra Rosskopfova, Jean Yannis Perentes, Hans-Beat Ris, Fabrizio Gronchi, Thorsten Krueger, Michel Gonzalez

**Affiliations:** 1 Division of Thoracic Surgery, Centre Hospitalier Universitaire Vaudois, Lausanne, Switzerland; 2 Division of Thoracic Anesthesiology, Centre Hospitalier Universitaire Vaudois, Lausanne, Switzerland

**Keywords:** Extracorporeal lung support, Tracheal resection, Carinal resection, Non-small cell lung cancer, Cardio-pulmonary bypass

## Abstract

Extracorporeal assistances are exponentially used for patients, with acute severe but reversible heart or lung failure, to provide more prolonged support to bridge patients to heart and/or lung transplantation. However, experience of use of extracorporeal assistance for pulmonary resection is limited outside lung transplantation. Airways management with standard mechanical ventilation system may be challenging particularly in case of anatomical reasons (single lung), presence of respiratory failure (ARDS), or complex tracheo-bronchial resection and reconstruction. Based on the growing experience during lung transplantation, more and more surgeons are now using such devices to achieve good oxygenation and hemodynamic support during such challenging cases. We review the different extracorporeal device and attempt to clarify the current practice and indications of extracorporeal support during pulmonary resection.

## Background

Locally advanced pulmonary cancers invading vital structure such as heart, great vessels, or carina cancer are generally considered as unresectable and incurable. Complete resection in healthy tissue may be compromised due to the proximity of the tumors to vital organs [1, 2]. Palliation with chemotherapy and/or radiotherapy is the principal means of treatment [1, 2]. Multimodality approach with combination of both chemotherapy and radiotherapy may downstage some patients with locally advanced pulmonary cancer. In addition, progress in anesthesia coupled with improved surgical techniques has helped redefine the limits of resection [3]. In highly selected patients with specific anatomic conditions, complete R0 resection for locally advanced tumor has been reported with prolonged survival and, on occasion, resulted in cure [4–6]. Conventional techniques frequently do not allow for complete resection of advanced pulmonary tumors invading the heart or great vessels. Complex cardiac resections or reconstructions, replacement of the thoracic aorta, or the common pulmonary artery can only be approached with cardiac arrest and total circulatory support by standard cardio-pulmonary bypass (CPB) [7–10]. In contrast, for tracheo-bronchial surgery or single lung situations, hemodynamic stability or cardiac arrest is not mandatory [5]. However, standard mechanical ventilation (jet-ventilation and/-or cross-field ventilation) may be insufficient to achieve good oxygenation in complex tracheo-bronchial resection and reconstruction [11]. Extracorporeal membrane oxygenation (ECMO) is a well-established technique for the management of respiratory or hemodynamic disturbance during lung transplantation [12]. Intra-operative use of veno-venous (VV) or veno-arterial (VA) ECMO allows good oxygenation in addition to removal of CO2 and may provide a complete ventilator support to complete tracheo-bronchial surgery or patient with single lung or presenting lung failure [13–18]. Technological advances in pumps, cannulae, and oxygenators and growing experience for thoracic surgeon during lung transplantation have led to the use of ECMO during conventional thoracic surgical procedure [13, 14]. Actually, ECMO experience for pulmonary resection is limited to the description of clinical cases without real agreement [18]. Pumpless interventional lung assist (Novalung) is an extracorporeal assistance which consists in a pumpless membrane ventilator providing low oxygenation and good carbon dioxide gas exchange and could be useful in selected situations [19, 20]. The aim of this review is to report current experience of the different extracorporeal device available for the thoracic surgeon and clarify the different indications.

### Cardio-pulmonary bypass

In case of NSCLC invading the heart or great vessels, conventional techniques allow a complete resection in only 30 to 40 % of such cases [21]. Complex cardiac resections or reconstructions, replacement of the thoracic aorta, or the common pulmonary artery can only be approached with a standard circulatory support by CPB. Few authors have reported their experience of resection of lung cancer with CPB [8, 22–28]. It has been estimated that less than 0.1 % of all thoracic resections are done by CPB [24].

Classical cardio-pulmonary bypass requires central cannulation by standard sternotomy and pericardiotomy. In different institutions, the introduction and the management of CPB is generally performed by cardiac surgeon. Normally, patients are placed on CPB with bi-caval venous cannulation and arterial return in the ascending aorta. Full systemic heparinization is mandatory with an ACT between 300 and 400 s and introduced before cannulation. Systemic hypothermia may be moderate (32°) or profound (18°) depending on the extension of resection and the vessels reconstruction. Cold-blood potassium cardioplegia is required to protect myocardium during the period of cardiac arrest, intra-cardiac resection, and reconstruction.

The main advantage of CPB is to provide a complete stability for gas exchange and hemodynamic support. This stability gives the possibility of complete inspection of infiltrated cardiac or vascular structure allowing for safe resections margins which can be controlled intra-operatively by frozen section. CPB may also promptly be inserted in case of emergent situation due to great vessels or heart lesions requiring reparation. However, CPB may present some inconvenient due to the necessity of full anticoagulation. Bleeding may be more frequent on peri- or post-operative course requiring more transfusion and re-operation for hemothorax [29]. Cardiac diseases have been reported to increase the risk for pulmonary complications following lung resections especially with prolonged use leading classically to pulmonary oedema, reperfusion injury, acute lung injury, or adult respiratory distress syndrome [22–25]. CPB may also activate inflammatory mediators or potentially spill tumor cells through the machine suction which could be responsible for decreased survival rate [30]. There is also a theoretical possibility of enhancement of metastasis due to the immunosuppression caused by the pump and blood transfusion [30–32].

There are been only five retrospectives studies of more than five patients since 2000 reporting the use of CPB for locally advanced pulmonary tumors or other intra-thoracic malignancies presenting invasion of heart and/or great vessels [22–25, 28] (Table 1). The indications varied from curative intention to palliative treatment. All series included restricted number of patients ranging from 7 to 19 patients. The indication to surgical therapy was made when no alternative treatment was available and based on the individual situation of the patient.

**Table 1:** Cardio-pulmonary bypass and pulmonary resection

Studies	Number	Primary tumor	Lung resection	Structure involved	Morbidity	Mortality (%)	Survival
Vaporciyan. 2002 [22]	19	Sarcoma (*n =* 9) Epithelial (*n =* 7) Others (*n =* 1)	PN (*n =* 5) Lobectomy (*n =* 3)	Great vessels (5 PA, 1 Ao, 5 IVC, 1 SVC) Heart (5 LA, 6 RA)	Overall 58 %: Pneumonia (37 %) Bleeding (21 %) Tracheotomy (16 %)	11	Curative intent (median): 64 months Palliative: 11 months
Park. 2004 [28]	10	Sarcoma (*n =* 7) Others (*n =* 3)	PN (*n =* 2) Lobectomy (*n =* 3)	Great vessels (5 SVC, 1 PA, 1 Aorta) Heart (4 LA, 2 RA)	Overall 50 % Re-operation 50 %	0	Complete resection (median: 33.3 months)
De Perrot. 2005 [23]	7	NSCLC (*n =* 7)	PN (*n =* 4) Lobectomy (*n =* 2) Carina (*n =* 1)	Great Vessels (2 SA, 1 Ao, 2 PA) Heart (2 LA) Carina 2	Overall (58 %) ARDS (1)	0	6/7 alive
Wiebe. 2006 [24]	13	Sarcoma (*n =* 8) NSCLC (*n =* 3) Others (*n =* 2)	PN (*n =* 9) Lobectomy (*n =* 4)	Great Vessels (PA 6, PV 3, Ao 1, SVC 1) Heart (LA 9, RA 3)	ALI (*n =* 4) Right heart failure (*n =* 1) MOF (*n =* 1)	15	Sarcoma 62 % at 5-year NSCLC 33 % at 5-year
Byrne. 2004 [25]	14	NSCLC (*n =* 7) Sarcoma (*n =* 5) Mesothelioma (*n =* 1) Thymic carcinoma (*n =* 1)	PN (*n =* 10) Lobectomy (*n =* 3) Wedge (*n =* 1)	Great vessels (PA 4, SVC 3, IVC 2 Heart (LA 3)	Low cardiac output syndrom (5/14) Re-operation for bleeding (3/14) Stroke (*n =* 1) Pulmonary edema (*n =* 1)	0	21 % at 5-year

*NSCLC* non-small cell lung cancer, *LA* left atrium, *RA* right atrium, *SVC* superior vena cava, *IVC* inferior vena cava, *Ao* aorta, *PA* main pulmonary artery or pulmonary trunk, *PN* pneumonectomy, *ALI* acute lung injury

Table summarizes the results of the current available series. In four series, there was a mixture of tumor types, mainly primary or metastatic sarcoma or pulmonary carcinoma and one series reporting only on pulmonary carcinoma. Concomitant lung resection consisted in pneumonectomy or lobectomy. Tumor invasion was restricted to one structure or invading multiple regions of the heart or great vessels such as aorta or pulmonary trunk. Heart invasion consisted only in left or right atrial invasion. There was no report on ventricular resection. In addition, one patient had a resection of the right and left coronary arteries and another patient required a coronary artery bypass grafting in the context of hemodynamically significant two-vessel coronary artery disease discovered on the pre-operative left heart catheterization. Emergent implementation of CPB was necessary in 33 % of patients (6/14) in the series of Byrne et al. required by injury of vital structure: superior vena cava (two patients), the inferior vena cava (two patients), or the pulmonary artery (two patients) [25]. In five of six patients, a right thoracotomy had been used, and emergent cannulation was achieved via the ascending aorta and right atrium or via bi-caval cannulation. In the remaining emergent patient, a left thoracotomy had been used, and cannulation was achieved via the descending thoracic aorta and the main pulmonary artery. In comparison, patients with planned surgery underwent central cannulation by sternotomy in five of the eight patients [25].

Sites of cannulation for CPB were mainly central with bi-caval and ascending aorta cannulation. However, some reported the use of peripheral access due to the extension of infiltration. The duration of CPB varied from 23 to 320 min. CPB achieved a complete cardiac arrest in the majority of patients with the use of severe hypothermia in one patient. In the series of De Perrot, four of the seven patients have induction chemotherapy or radio-chemotherapy [23]. Complete resection was finally reported in almost 80 % of patients with 5-year survival rate varying between 33 and 62 % depending on the primary tumor type. Incomplete R2 resection was achieved in some patient for palliative situation and could however alleviate good palliation for bulky tumor with median survival of 11 months [28].

Overall 30-day mortality ranged from 0 to 15 %. Overall morbidity was reported in more than 50 % of patients consisting in pneumonia or acute lung injury. Park reported that 50 % of patients requiring re-operation for bleeding.

The use of CPB does not appear to increase the risk of cancer dissemination with patient presenting long-term survival. Moreover, several series have reported patients with aortocoronary bypass surgery in combination with pulmonary surgery during the same operative procedure with good early and long-term results despite the use of CPB [10, 33].

Based on these different series, no conclusion can be drawn. In fact, there is actually no standard approach to this complex situation. All existing studies showed different techniques of CPB, different extension of pulmonary resection (lobectomy or pneumonectomy), different sites of infiltration by the tumor (atrium, aorta, or pulmonary trunk), types, and stages of tumors. Nevertheless, CPB is a feasible option of surgical therapy with or without neo-adjuvant treatment for pulmonary or mediastinal malignancies invading cardiac structure and/or great vessels. The choice of incision should be based first on whether complete surgical resection, after which the choice of cannulation sites should follow. The support of CPB may increase the rate of a complete resection and may improve long-term survival in highly selected patients provided that patient assumes the risk of high morbidity and re-operation rate. Furthermore, the emergent institution of CPB for the repair of injury to a vascular structure during pulmonary resection is lifesaving and effective.

### Extracorporeal membrane oxygenation

Extracorporeal membrane oxygenation (ECMO) represent a form of artificial circulatory and respiratory support used in the cases of respiratory or cardiac failure as a bridge to restoration of the function or to further lung transplantation. The published experience with this specific device in the mean of general thoracic surgery is limited except for the ARDS, lung transplantation, and neonatal-pediatric surgery for the management of different congenital pathologies of the airways [12, 15, 16, 34].

ECMO use the concept of extracorporeal circulation with the use of non-occlusive centrifugal pump and oxygenator which is responsible for enrichment of O_2_ and elimination of CO_2_. A thermostat can be introduced to the circuit to modulate the temperature. There is two basic form of ECMO: veno-arterial and veno-venous. These techniques assistance may be introduced peripherally or centrally depending on the specific indications [34]. ECMO may provide optimal oxygenation with a partial or total circulatory support. In comparison with classical CPB, ECMO system may present some advantages: the ECMO system needs theoretically low anticoagulation (ACT: 160–200) with a low dose of heparin (500–1000 UI) at the moment of the insertion of cannula provided that the cannula are heparin-coated; the risk of thrombosis of the ECMO system is very low during the short time of thoracic operation. In case of important bleeding, anticoagulation may be omitted. Others consider also that if ECMO flow is more than 3l, heparin can be dispensed during intra-operative use only [18]. There is no theoretical risk of tumor cell dissemination due to closed system devoid of cardiotomy suction during CPB. Moreover, ECMO may present to surgeon a clean operative field without disturbing line when cannula are introduced peripherally and stability of cardio-respiratory function during heart manipulation [13]. ECMO may be maintained post-operatively in case of pulmonary edema. ECMO may be also switched from veno-arterial to veno-venous ECMO to obtain a protective lung ventilation avoiding pressure on bronchial or tracheal sutures line in case of mechanical ventilation with high volumes. Finally, in case of emergent situation, it is possible to convert VA ECMO into conventional CPB by adding a venous reservoir connected to the ECMO device.

#### Veno-arterial ECMO

Veno-arterial (VA) ECMO can support either the cardiac and respiratory functions with good gas exchange and cardiac hemodynamic support. This type of assistance is recommended if the respiratory and cardiac support is required. When cannula is inserted peripherally, the sites of venous cannulation may be jugular interior vein or femoral vein (17 to 19 French) to gain access to right atrium. The oxygenated blood to the arterial circulation is then re-injected into the femoral or axillary artery. The insertion may be surgical or percutaneous provided that femoro-femoral ECMO is the most used in emergency situation due to the facility of vascular access in the groin region. In neonatal patient, carotid artery has been described as arterial cannulation. When central cannulation is decided or required, the venous cannula is inserted directly into the right atrium and the arterial cannula positioned into the ascending aorta. The complications of veno-arterial ECMO are mainly related to the vascular access with potential arterial dissection at the site of cannulation, or acute ischemia of the limb or late arterial stenosis. A reperfusion cannula may be inserted distally and connected to the arterial one in order to bring oxygenated blood to the lower limb through superficial femoral artery. Myocardial or brain hypoxemia may be encountered when the heart is still ejecting non-oxygenated blood while the lung is not ventilated, with hypoxic blood from the left ventricle entering into the coronary and cerebral circulation. Harlequin syndrome is characterized by competition of blood flow between hypoxic blood ejected by the left ventricle and the blood oxygenated from the peripheral ECMO with insufficient retrograde flow which could be responsible of brain or myocardial damage [34]. The patient should be correctly monitored with captor of blood saturation on the upper part body, and situation promptly recognized and required change of the type of ECMO.

#### Veno-venous ECMO

Veno-venous (VV) ECMO supports the lung only for oxygenation and CO_2_ extraction. VV ECMO may be used for elective cases in the absence of cardiac failure or cardiac instability. The venous blood is aspired to the reservoir, and after the passage by the oxygenator, the blood is re-injected by a pump back to the venous system. Unlike VA ECMO with potential arterial complications, VV ECMO needs only venous cannulation which may be inserted in femoro-jugular or jugulo-femoral. Recently, there has been the development of a double lumen cannula inserted in the right jugular vein [35]. A single site cannulation is only needed and be inserted easily in the intensive care before the operation or in the operating room. The double lumen cannula may be maintained in post-operative course in case of post-operative ARDS, and patient may be extubated and early mobilized. The risk of re-circulation is theoretically decreased. This cannula should be positioned under both control of transoesophageal echocardiography and fluoroscopy to avoid the risk of right ventricular perforation. VV ECMO may fully support gas exchange and replace ventilation leading to long period of apnea. Myocardial and brain oxygenation is better maintained with oxygenated blood getting directly through the systematic circulation. Complications related to VV ECMO are thromboembolic venous disease, superior vena cava syndrome, and re-circulation [36].

#### Indications of ECMO

During thoracic surgery, airway management may be sometimes problematic. The balance need to be obtained between sufficient surgical exposure and adequate control of ventilation. This holds especially true for tracheo-bronchial resection and reconstruction where selective ventilation is required. Ventilation can be achieved by two methods: the cross field periodic intubation of the main bronchus alternating periods of apnea with ventilation and jet ventilation through a small catheter passed through the field into the contralateral main bronchus [5]. These two techniques are generally sufficient for carinal resection and reconstruction. De Perrot reported only 3 of 119 patients requiring emergency use of CPB during carinal resection due to intra-operative edema of the contralateral lung (*n =* 2) and major bleeding from the right main pulmonary artery. However, these techniques may present some limitations particularly in case of severe tracheo-carinal stenosis where pre-operative intubation is not feasible. CO_2_ removal during jet ventilation can be troublesome in patients with severe COPD or obesity. Jet ventilation may also create barotraumatism in the contralateral lung or may potentially cause spilling of mucosal tumor cell spread. Furthermore, the different tube in the operating field may impair the surgical access and visibility leading to tension on anastomosis during complex reconstruction. Good cooperation with anesthesiologic team is required for tube placement and replacement which can lead to period of long desaturation. Moreover, lack of hemodynamic stability may appear if extended retraction maneuver of the heart are needed.

In case of single lung procedure after previous pneumonectomy, ventilation can be managed by endotracheal ventilation with high-flow oxygen through a small catheter with tidal volume. However, this simple technique may produce severe hypercapnic acidosis, and duration of the technique is generally limited to 30 min. Finally, in case of severe respirators failure, the surgeon needs to operate with ventilated lung, but this technique does not allow to perform complex surgical procedure.

The use of ECMO has been initially reported in the pediatric population for the management of different congenital tracheal pathologies requiring complex reconstruction with concomitant repair of artery sling or patent ductus arteriosus [37–40]. These experiences in infants allowed to determine potential implication for the use of ECMO for adults: the use of ECMO could be beneficial as a bridge to definite tracheal surgery; the use of ECMO resulted in better visualization at the surgical site without the need for endotracheal tubes and aggressive ventilation technique. Finally, the ECMO allowed to perform complex operative procedure under stable cardio-respiratory conditions. In adult population, ECMO is routinely used for the management of ARDS, severe thoracic trauma, or during lung transplantation [34]. Thoracic surgeon is now more and more familiar with the use of ECMO especially in the era of lung transplantation [12]. ECMO may be inserted as a bridge during the waiting period, during the operation in case of cardio-pulmonary instability, or in the post-operative course in case of primary graft dysfunction. The use of ECMO during thoracic surgery other than lung transplantation has been initially reported in 1996 by Horita who performed two successful resections and reconstruction of the carina under VV ECMO [41]. Since then, others reported use of ECMO for complex tracheo-carinal resection and reconstruction, during single lung procedure after previous pneumonectomy or during ARDS: carinal resection [14, 42], mediastinal tumor resection with compression of the trachea [43], single-lung segmentectomy [44], tracheo-bronchial repair for traumatic injury [45, 46], emphysemateous bulla resection in single lung, or limited resection of the lung (wedge or segmentectomy) for aspergillosis or lung abscess during ARDS [47, 48].

Lang et al. reported their experience in two recent series with the use of veno-arterial ECMO for complex tracheo-carinal resection for central tumor [13, 14] (Table 2). Carinal resection and reconstruction was associated in some patients with pulmonary resection (pneumonectomy or lobectomy). Cannula was implanted both in peripheral or central position depending on the planned surgical approach. The mean time of ECMO was 110 and 113 min. Interestingly, they do not report complication related to the ECMO device or cannula access with no bleeding or arterial complications. They could achieve a complete R0 resection in more than 80 % with an interesting 5-year survival rate of 56 %. They described total cardio-pulmonary stability and clean operative field allowing for safe resection and reconstruction. They used low anticoagulation with heparin with ACT below 200 s. Recently, Rinieri et al. reported a national review of the use of ECMO as respiratory support in thoracic surgery excluding lung transplantation and lung resection for tumors invading the great vessels and/or the left atrium with a questionnaire in 34 thoracic centers in France taking into account years 2009–2012 [18]. There were 17 centers that applied ECMO in 36 patients. The type of ECMO and type of resection are resumed in Table 3. Total respiratory support with VV (*n =* 12) or VA (*n =* 16) with interruption of ventilation was required in 28 patients for tracheo-bronchial (*n =* 23) and single-lung (*n =* 5) procedures. Time off ventilation median duration during ECMO was 78 and 65 min for VV and VA ECMO, respectively. ECMO morbidity consisted in bleeding requiring re-operation in six patients (17 %) and two cannulation-related complications (6 %). Overall 30-day mortality was 17 % (*n =* 6).

**Table 2:** Extracorporeal device (ECMO veno-arterial and/or veno-venous) and pulmonary resection for pulmonary cancer

Series	Number	Primary tumor	Pulmonary procedure	Type of ECMO and duration	Morbidity (ECMO)	Outcome
Lang 2011 [14]	9	NSCLC (*n =* 7) Carcinoid (*n =* 1) Sarcoma (*n =* 1)	Complex tracheo-bronchial resection (*n =* 6) associated with pneumonectomy (*n =* 4) or bilobectomy (*n =* 1) Resection descending aorta (*n =* 2) Resection inferior vena cava (*n =* 1)	Central (*n =* 4) Peripheral (*n =* 4) Combined (*n =* 1) Mean time 110 min (range 40 to 135)	- Lymphatic fistula to groin - No bleeding - No vascular thrombosis	Complete resection 8/9 (89 %) Mortality 1/9 (hepatic necrosis after IVC resection)
Lang 2014 [13]	10	NSCLC (*n =* 7) Carcinoid (*n =* 2) Adenoid cystic (*n =* 1)	Carina (*n =* 6) Sleeve lobectomy (*n =* 3) Pneumonectomy (*n =* 1)	Central (*n =* 7) Peripheral (*n =* 3) Mean time 113 min (range 70–135)	- No bleeding - No vascular thrombosis	Complete resection 8/10 (80 %) 5-year survival 56 %
Rinieri 2014 [18]	36	NA	Tracheo-carinal resection (*n =* 21) Left main bronchus resection (*n =* 2) Single-lung surgeries (*n =* 5) Pulmonary resection (*n =* 5) Thoracic trauma (*n =* 2)	Veno-venous (*n =* 20) Mean time 78 min VA peripheral (*n =* 10) VA central (*n =* 6) Mean time 65 min	Bleeding with re-operation (*n =* 6) Bleeding cannulation site (*n =* 1) Inguinal infection (*n =* 1) No thrombosis or ischemia limb	30-day mortality 17 % (*n =* 6)
Redwan 2015 [49]	9	NSCLC (*n =* 6) Pulmonary metastases (*n =* 2) COPD (*n =* 1)	Segmentectomy (*n =* 3) lobectomy with bronchial and vascular anastomoses (*n =* 1) VATS lobectomy (*n =* 2) Left-sided carinal pneumonectomy (*n =* 1) Metastasectomy (*n =* 2)	Veno-venous (*n =* 9) Mean time 19 min (range: 71–184)	Pneumonia (*n =* 5) Tracheostomy (*n =* 1) Acute cardiac failure (*n =* 1)	30-day mortality 11 % (*n =* 1)

*NA* not available

**Table 3:** Summary of different extracorporeal devices

	Inconvenient	Longer operations ➢ Cardiac disease have been reported to increase the risk for pulmonary complications following lung resections especially with prolonged use (pulmonary oedema) ➢ Full anticoagulation (ACT >300 s) ➢ Bleeding (transfusion, re-operation) ➢ Activation of inflammatory mediators ➢ Potential danger of tumor cell spilling through the machine suction
Cardio-pulmonary bypass	Indication	➢ Total pulmonary support (CO_2_ extraction and O_2_) hemodynamic stability and possibility of cardiac arrest
	Advantage	➢ Complete inspection of infiltrated cardiac or vascular structures allowing for safe resections margins ➢ Intra-operative microscopic control of complete resection ➢ Emergent institution in case of great vessels lesion
	Inconvenient	Longer operations ➢ Cardiac disease have been reported to increase the risk for pulmonary complications following lung resections especially with prolonged use (pulmonary oedema) ➢ Full anticoagulation (ACT >300 s) ➢ Bleeding (transfusion, re-operation) ➢ Activation of inflammatory mediators ➢ Potential danger of tumor cell spilling through the machine suction
Veno-arterial ECMO	Indication	➢ Total pulmonary support (CO2 extraction and O2) and hemodynamic stability
	Advantage	➢ No risk of tumor cell dissemination (closed system devoid of cardiotomy suction) ➢ Low anticoagulation (ACT:160-200 s). Cannulae are heparin-coated ➢ Clean operative field without disturbing line ➢ Stability of cardiorespiratory function during heart manipulation ➢ Switch VA to VV ECMO: protective lung ventilation (no pressure on sutures in case of mechanical ventilation with high volumes). VA ECMO can be quickly converted into conventional CPB in case of cardiovascular wound
	Inconvenient	➢ Arterial dissection/thrombosis ➢ Acute ischaemia of limb ➢ Myocardial or brain hypoxaemia
Veno-venous ECMO	Indication	➢ Total pulmonary support (CO_2_ extraction and O_2_)
	Advantage	➢ Useful for elective cases if no cardiac failure or cardiac morbidity ➢ No arterial cannulation with no risks of arterial injury ➢ Better myocardial oxygenation ➢ Possibility to maintain post-operatively in case of pulmonary oedema
	Inconvenient	➢ Thromboembolic venous disease ➢ Recirculation ➢ Superior cava syndrome
Interventional lung assist (Novalung)	Indication	➢ Partial pulmonary support (CO_2_ extraction, low oxygenation)
	Advantage	➢ Pumpless membrane ventilator ➢ Low anticoagulation ➢ Apnea possible with passive endotracheal oxygenation ➢ Peripheral access by percutaneous cannulation
	Inconvenient	➢ Vascular access complications (dissection, thrombosis) ➢ Only part of the cardiac output (1–2 L/min) for extracorporeal gas exchange. (Low capacity of oxygenation) ➢ Adequate mean arterial blood pressure is mandatory. It may not be used as: o Low cardiac output o Impaired left ventricular function o High dose catecholamine administration

**Fig. 1:**
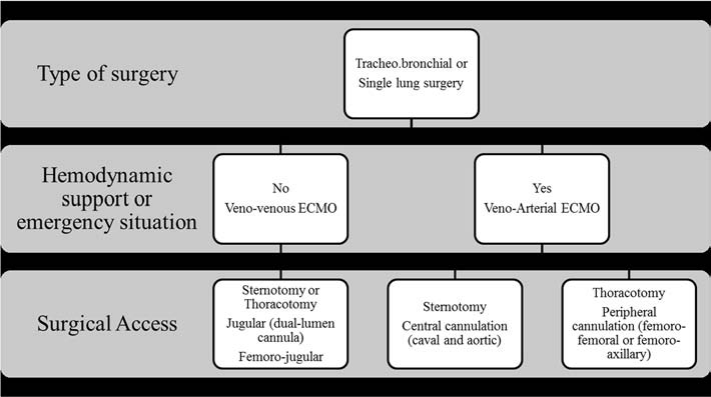
algorithm for insertion of ECMO in case of elective or emergent thoracic surgery (adapted from Rinieri et al. [18]

Based on their report, Rinieri et al. proposed a simple algorithm for the use of ECMO based on the degree of emergency, the necessity of hemodynamic support, and the surgical access (Fig. 1) [18]. In case of emergent situation (major bleeding or cardiac instability), VA ECMO should be privileged either by femoral cannulation if the patient lies on supine position or in central position if surgical access allows central cannulation. Hemodynamic instability due to cardiac failure, pulmonary hypertension, or major cardiac retraction necessitates VA ECMO for circulatory support. In case of tracheo-carinal resection, VV ECMO by dual-lumen cannula inserted in the right jugular vein seems guaranteed enough respiratory support in the absence of hemodynamic disturbance. Redwan et al. reported their experience with veno-venous ECMO for major resection. VV ECMO allowed apnea for 40 min, time necessary for resection and reconstruction of complex procedure such as left sided carinal pneumonectomy [49].

In conclusion, the use of ECMO remains exceptional and reported in small collectives of patients. However, VV and VA ECMO may be useful as total respiratory support in complex tracheo-bronchial surgery or single-lung surgery where conventional ventilation technique is not feasible. The choice between VA and VV ECMO depends on the need of circulatory support, surgical access, and current practice.

### Pumpless interventional lung assist (Novalung)

Pumpless interventional lung assist (iLA) consists of a pumpless membrane oxygenator that is driven by the difference in arterial and venous blood pressure [19]. Only part of the cardiac output (1–2 L/min) is accessible for extracorporeal gas exchange reason why this system allows only good CO_2_ removal, but low oxygenation. As the system is pumpless with an arterio-venous configuration, an adequate mean arterial blood pressure is mandatory. The preferred access sites are the femoral vessels (venous 19F /arterial 17F) by percutaneous cannulation using Seldinger’s technique. All the components of the system are heparin coated, and generally, it is recommended a single bolus injection of 500 to 1000 units of heparin before introduction of the cannula.

Wiebe et al. reported ten patients who underwent thoracic surgical procedure under the Novalung device [20]. Six of them required Novalung on emergent situation. Five patients required lung surgery for single-lung situation (wedge, decortication, bronchial repair, and esophageal resection). The average flow of Novalung was 1.58 L/min allowing adequate CO_2_ extraction and correction of the pH in all patients. In addition, oxygenation was managed with administration of oxygen 100 % in the trachea via endotracheal small bore catheter at low pressure (3–10 mmHg) allowing apneic oxygenation in four patients up to 60 min. They reported retroperitoneal hematoma after percutaneous removal of arterial cannula related to device.

Although Novalung is capable of controlling any hypercapnic complications, its capacity for oxygenation is limited in comparison with ECMO. Furthermore, this pumpless extracorporeal lung assist required good cardiac function and should cautiously used in case of low cardiac output, impaired left ventricular function or administration of high dose of catecholamine. Moreover, complications have been reported in 10 to 24 % related to access device [50]. In conclusion, the Novalung device could be an alternative in case of pure hypercapnic situations without impairment of oxygenation or hemodynamic stability. Hypoxemic situation after implementation can be managed with adjunction of low-pressure oxygen allowing for period of apnea reaching up 1 h in selected cases.

## Conclusions

Cardio-pulmonary bypass, extracorporeal membrane oxygenation, and pumpless extracorporeal lung assist have their place in the general thoracic surgery considering that a growing number of patients will benefit complex resection due to the advance in oncological treatment and improvement in surgical and anesthesic techniques. The choice of the right device depends on the type of surgery and the type of cardio-respiratory status. Carefully selected patients with tumor involvement of the heart or great vessels should be resected on CPB with acceptable mortality and morbidity. It allows achievement of good palliation and long-term survival in a few. VV and VA ECMO can serve as total respiratory support in complex tracheo-bronchial surgery or single lung surgery where conventional ventilation techniques are not feasible. VA ECMO is a choice in patients where cardiac support is needed compared to VV ECMO that sustains respiratory functions only. Pumpless extracorporeal lung assist device with passive endotracheal oxygenation could be an alternative for patient without hypoxemic or hemodynamic instability. A combined and well-coordinated effort among the general thoracic surgeon, the cardiac surgeon, the perfusionist, and the anesthesiologist is required for a successful outcome. All those methods are feasible but are reserved to centers with extensive experience and practice handling of those systems.

## Abbreviation

CPB: cardio-pulmonary bypass
ECMO: extracorporeal membrane oxygenation
VA: veno-arterial
VV: veno-venous
